# Polymorphisms in the *epidermal growth factor receptor *gene and the risk of primary lung cancer: a case-control study

**DOI:** 10.1186/1471-2407-7-199

**Published:** 2007-10-24

**Authors:** Jin Eun Choi, Sun Ha Park, Kyung Mee Kim, Won Kee Lee, Sin Kam, Sung Ick Cha, Chang Ho Kim, Young Mo Kang, Young-Chul Kim, Sung Beom Han, Tae Hoon Jung, Jae Yong Park

**Affiliations:** 1 Department of Biochemistry, School of Medicine, Kyungpook National University, Dong In 2Ga 101, Daegu, 700-422, South Korea; 2 Cancer Research Institute, Kyungpook National University Hospital, Samduk 2Ga 50, Daegu, 700-412, South Korea; 3 Department of Internal Medicine, Kyungpook National University Hospital, Samduk 2Ga 50, Daegu, 700-412, South Korea; 4 Department of Preventive Medicine, School of Medicine, Kyungpook National University, Dong In 2Ga 101, Daegu, 700-422, South Korea; 5 Department of Internal Medicine, Chonnam National University Medical School, Hag 1Dong 5, Gwanguj 501-746, South Korea; 6 Department of Internal Medicine, School of Medicine, Keimyung University, Dongsan Dong 194, Daegu, 700-712, South Korea

## Abstract

**Background:**

Polymorphisms in Epidermal Growth Factor Receptor (*EGFR*) gene may influence EGFR production and/or activity, thereby modulating susceptibility to lung cancer. To test this hypothesis, we investigated the association between polymorphisms in the *EGFR *gene and the risk of lung cancer in a Korean population.

**Methods:**

We first examined the frequencies of 39 candidate polymorphisms in the *EGFR *gene in 27 healthy Korean individuals. After then, we genotyped five polymorphisms (127378C>T, 142285G>A, 162093G>A, 181946C>T and 187114T>C) that have variant allele frequencies greater than 10%, in 582 lung cancer patients and in 582 healthy controls.

**Results:**

Of the 5 polymorphisms, the 181946C>T genotype distribution was significantly different between the cases and controls (*P *= 0.04). Compared with the 181946 CC + CT genotype, the 181946 TT genotype was associated with a significantly decreased risk of lung cancer (adjusted OR = 0.63, 95% CI = 0.45–0.88, *P *= 0.007). When the analyses were stratified by smoking status, the protective effect of the TT genotype was statistically significant in ever-smokers (adjusted OR = 0.59, 95% CI = 0.41–0.86, *P *= 0.007), but not in never-smokers (adjusted OR = 0.89, 95% CI = 0.45–1.75, *P *= 0.73; *P *= 0.08, test for homogeneity). Consistent with the results of the genotyping analysis, the CGGCT haplotype with the 181946C allele was associated with a significantly increased risk of lung cancer compared to the CGGTT haplotype carrying the 181946T allele (adjusted OR = 1.50, 95% CI = 1.09–2.07, *P *= 0.012 and Bonferroni corrected *P*-value = 0.048).

**Conclusion:**

These results suggest that the *EGFR *polymorphisms, particularly the 181945C>T polymorphism, could be used as markers for the genetic susceptibility to lung cancer.

## Background

The development and progression of lung cancer is a multi-step process characterized by the accumulation of multiple genetic and epigenetic alterations, that results in perturbations of cell-regulatory and growth-control pathways [1,2]. The epidermal growth factor receptor (EGFR)-driven pathway is known to be one of the known important oncogenic signang cascades in lung cancer [2-4].

The EGFR is a tyrosine kinase receptor that belongs to the ERBB family, and it mediates the intracellular effects of growth factors such as EGF, transforming growth factor α and neuregulins. The activation of EGFR via overexpression of the receptor and/or ligands or its structural alteration, affects a number of processes important to cancer development and progression, including cell proliferation, apoptosis, angiogenesis, and metastasis [5-7].

Single nucleotide polymorphisms (SNPs) are the most common sources of human genetic variation, and they may contribute to an individuals' susceptibility to cancer. Several studies have demonstrated that some variants affect either the expression or activities of various enzymes, and that they are therefore associated with the risk of cancer development [8-11]. Several polymorphisms in the *EGFR *gene have been reported [12-16] and deposited into public databases [17]. Although the functional effects of these polymorphisms have not yet been fully elucidated, we hypothesized that some of these variants may have an effect on EGFR expression or activity, and therefore may play a role in modulating the susceptibility to lung cancer. To test this hypothesis, we performed a case-control study to investigate the association between *EGFR *genotypes/haplotypes and the risk of lung cancer.

## Methods

### Identification and selection of polymorphisms

Among the candidate polymorphisms in the *EGFR *gene, we initially captured 39 SNPs in the promoter region, all exons including intron-exon boundaries (10 bp of the introns on either side) and the 3'-UTR of the gene because variants in these regions are most likely to affect gene function (Table 1). We then examined the frequencies of the captured SNPs in a preliminary study that included 27 healthy controls and 27 lung cancer cases. Among the 39 captured SNPs, seven SNPs [127378C>T (rs2072454), 142232C>T (rs17336800), 142285G>A (rs11543848), 151904T>A (rs17290169), 162093G>A (rs10251977), 181946C>T (rs2293347), and 187114T>C (rs884225)] had minor allele frequencies greater than 10% in the 54 subjects. The 142232C>T and 151904T>A were completely or near completely linked with the 142285G>A. Thus five SNPs (127378C>T, 142285G>A, 162093G>A, 181946C>T and 187114T>C) were chosen for the association study.

**Table 1:** Known and candidate polymorphisms in the *EGFR *gene

			Variant allele frequency^†^				
			
Region^†^	Nucleotide (amino acid) change^†^	SNP ID^†^	Korean (27 controls/27 cases)	Global	Caucasian	Asian	African American
Promoter	-1433C>T	rs17335689	0.00/0.00	0.017	0.00	0.00	0.042
	-1298G>A	rs17335696	0.00/0.00	0.006	0.00	0.00	0.042
	-1247G>A	rs6593197	0.00/0.00	-	0.00	0.00	0.104
	-1225G>A	-	0.00/0.00	-	0.023	0.00	0.00
	-759C>A	rs759171	0.00/0.00	0.101	0.136	0.00	0.146
	-646G>A	-	0.00/0.00	-	0.00	0.00	0.042
	-615C>G	rs13228815	0.00/0.00	-	-	-	-
	-540G>A	-	0.02/0.00	-	0.00	0.024	0.00
	-482C>A	rs17335710	0.00/0.00	0.006	0.00	0.00	0.063
	-216G>T	rs712829	0.02/0.04	0.222	0.318	0.071	0.292
	-191C>A	rs712830	0.00/0.00	0.078	0.136	0.00	0.00
Intron 1	169G>T	rs17335738	0.00/0.00	0.101	0.114	0.00	0.114
	2028G>A	-	0.07/0.07	-	0.023	0.095	0.042
Exon 3	124080G>A (R98Q)	rs17289589	0.00/0.00	0.006	-	-	-
Exon 4	**127378C>T (N158N)**	rs2072454	0.37/0.32	0.415	0.500 (0.42)^‡^	0.320	0.457
	127417C>T (D171D)	rs17289686	0.00/0.00	0.011	-	-	-
	127435G>A (S177S)	rs17336437	0.00/0.00	0.028	-	-	-
Intron 4	127473G>A IVS4+10)	rs7801956	0.00/0.02	0.051	0.070	0.039	0.008
Exon 7	134783C>G (P266R)	rs17336639	0.00/0.00	0.006	-	-	-
Exon 8	136584C>T (C307C)	rs17289893	0.00/0.00	0.011	-	-	-
Exon 9	137368G>A (P373P)	rs2302536	0.00/0.00	0.006	0.00	0.007	-
Exon 12	140880G>A (A439A)	rs17290005	0.00/0.00	0.022	-	-	-
Exon 13	142232C>T (G503G)	rs17336800	0.39/0.41	0.006	-	-	-
	**142285G>A (R521K)**	rs11543848	0.39/0.41	0.289	0.250 (0.26)^‡^	0.500	0.109
Exon 14	144456T>C (G544G)	rs17290103	0.00/0.00	0.022	-	-	-
Exon 15	146055T>C (V592A)	rs28384375	0.00/0.00	-	-	-	-
	146068G>A (P596P)	rs17290162	0.00/0.00	0.017	-	-	-
	146119C>T (A613A)	rs17290169	0.00/0.00	0.08	- (0.05)^‡^	-	-
	146151G>T (C624F)	rs28384376	0.00/0.00	-	-	-	-
Exon 16	151904T>A (T629T)	rs17337023	0.37/0.41	0.456	- (0.36)^‡^	- (0.446)§	-
Exon 17	153806G>A (V674I)	rs17337079	0.00/0.00	0.006	-	-	-
Exon 18	154737G>T (G719C)	rs28929495	0.00/0.00	-	-	-	-
Exon 20	**162093G>A (Q787Q)**	rs10251977	0.07/0.13	0.427	0.604 (0.48)^‡^	0.146	0.457
Exon 21	172480C>T (R836R)	rs17518376	0.00/0.00	0.011	0.083 (0.07)^‡^	0.00	0.00
Exon 23	179447T>C (T903T)	rs1140475	0.07/0.06	0.111	0.117 (0.13)^‡^	0.06	0.017
Exon 24	181074C>G (R962G)	rs17337451	0.00/0.00	0.06	-	-	-
Exon 25	181927A>C (H988P)	rs17290699	0.00/0.00	0.006	-	-	-
	**181946C>T (D994D)**	rs2293347	0.43/0.35	0.197	0.136	0.286	0.053
3'UTR	**187114T>C**	rs884225	0.37/0.31	0.869	0.150	0.475	0.025

^† ^Information about SNPs, SNP ID and frequencies of variant alleles in other ethnic populations were obtained from NCBI database . In the reference sequence (GenBank accession no. NT_033968), the translation start site was counted as +1. In the cases of polymorphisms in the promoter and intron 1 (*i.e*., from -1433C>T to 2028G>A), frequencies of variant alleles in Caucasians, Asians and African Americans were obtained from Ref. 15.

^‡ ^Data in the parenthesis were obtained from Ref. 14.

^§ ^Datum in the parenthesis was obtained from Ref. 24.

### Study population

This case-control study included 582 lung cancer patients and 582 healthy controls (Table 2), and the details of the study population have been described previously [18,19]. In brief, the eligible cases included all patients who were newly diagnosed with primary lung cancer between January 2001 and June 2002 at Kyungpook National University Hospital, Daegu, Republic of Korea. There were no age, gender, histological, or stage restrictions, but patients with a prior history of cancer were excluded from the study. The cases included 270 (46.4%) squamous cell carcinomas, 205 (35.2%) adenocarcinomas, 97 (16.7%) small cell carcinomas, and 10 (1.7%) large cell carcinomas. The control subjects were randomly selected from a pool of healthy volunteers who visited the general health check-up center at Kyungpook National University Hospital during the same period. A total of 3065 (1598 males and 1467 females) of 5578 healthy subjects agreed to participate in this study (participation rate, 54.9%). Compared with subjects that refused to participate, enrolled subjects showed similar sex (% of male, 52.5% versus 52.1%; *P *= 0.80) and age (52.2 ± 11.4 versus 52.1 ± 11.3; *P *= 0.80) distributions. From 3065 healthy volunteers, we randomly selected 582 control subjects that were frequency-matched (1:1) to the cases based on sex and age (± 5 years). All of the cases and the controls were ethnic Koreans and they resided in Daegu City or the surrounding regions. This study was approved by the institutional review board of the Kyungpook National University Hospital, and written informed consent was obtained from each participant.

**Table 2:** Characteristics of the study population

Variable	Cases (n = 582)	Controls (n = 582)
Age (years)	61.3 ± 9.4	60.2 ± 9.6
Sex		
Male	467 (80.2)^a^	467 (80.2)
Female	115 (19.8)	115 (19.8)
Smoking status^b^		
Current	387 (66.5)	297 (51.0)
Former	85 (14.6)	147 (25.3)
Never	110 (18.9)	138 (23.7)
Pack-years^c^	40.0 ± 17.7	34.1 ± 17.8

^a ^Numbers in parenthesis, percentage.

^b ^*P *< 0.001.

^c ^In current and former smokers, *P *< 0.001

### EGFR genotyping

Genomic DNA was extracted from peripheral blood lymphocytes by proteinase K digestion and phenol/chloroform extraction. The *EGFR *127378C>T, 142285G>A (R521K), 162093G>A (N158N), 181946C>T (Q787Q) and 187114T>C genotypes were determined using a PCR-RFLP assay. PCR primers were designed based on the GenBank reference sequence (accession no. NT_033968). The PCR primers for 127378C>T, 142285G>A, 162093G>A, 181946C>T and 187114T>C polymorphisms were 5'-ATTGCGGGACTCTTGTTCGC-3' (forward) and 5'-CGCCACTGGATGCTCTCCG (mutated A→G)C-3' (reverse); 5'-TCCCTGCTCTGTCACTGACT-3' (forward) and 5'-T AACAACAACCTGGAGCCTT-3' (reverse); 5'-TGCCTCACCTCCACCGTGG (mutated C→G)A-3' (forward) and 5'-GCACGCACACACATATCCCC-3' (reverse); 5'-ATTGG CAAACACACAGGCAC-3' (forward) and 5'-CTGCTGAAGAA GCCCTGCTG-3' (reverse); and 5'-AGAAACGGAGGGGATGGAAT-3' and 5'-AGGTATTCCACATTCT CAGC-3' (reverse), respectively. PCR reactions were performed in a 20 μl reaction volume containing 100 ng genomic DNA, 10 pM of each primer, 0.2 mM dNTPs, 10 mM Tris-HCl (pH 8.3), 50 mM KCl, 2.5 mM MgCl_2_, 5% DMSO and 1 unit of Taq polymerase (Takara Shuzo Co., Otsu, Shiga, Japan). The PCR cycle conditions consisted of an initial denaturation step at 95°C for 5 min followed by 35 cycles of 30 s at 94°C 30 s at 58°C for 127378C>T and 187114T>C, 54°C for 142285G>A, 57°C for 162093G>A, and 56°C for 181946C>T; 30 s at 72°C; and a final elongation at 72°C for 10 min. The PCR products were digested overnight with the appropriate restriction enzymes (New England BioLabs, Beverly, MA, USA) at 60°C (127378C>T) or 37°C (162093G>A, 142285G>A, 181946C>T and 187114T>C). The restriction enzymes for 127378C>T, 142285G>A, 162093G>A, 181946C>T and 187114T>C genotypes were *BstU*I, *BstN*I, *Ban*II, *Mly*I, and *Aci*I, respectively. The digested PCR products were resolved on 6% acrylamide gel and stained with ethidium bromide for visualization under UV light. To ensure quality control, the genotyping analysis was performed "blind" with respect to case/control status. About 10% of the samples were randomly selected to be genotyped again by a different investigator, and the results were 100% concordant. Information about all SNPs, SNP ID and allele frequency was obtained from the NCBI homepage [17]. In the reference sequence, the translation start site was counted as +1.

### Statistical analysis

The cases and controls were compared using the Student's *t*-test for continuous variables and a χ^2 ^test for categorical variables. Hardy-Weinberg equilibrium was tested using a goodness-of-fit χ^2 ^test with one degree of freedom to compare observed genotype frequencies with expected genotype frequencies among the subjects. The strength of LD between pairs of polymorphisms was measured by HaploView [20]. The haplotypes and their frequencies were estimated based on a Bayesian algorithm using the Phase program [21]. Conditional logistic regression analysis was used to calculate odds ratios (ORs) and 95% confidence intervals (CIs) for overall lung cancer, with adjustment of pack-years of smoking (as a continuous variable). In addition to the overall association analysis, we performed a stratified analysis by age (median age, ≤ 62 years/>62 years), gender, smoking status, cigarette exposure level (median pack-years of smoking in ever-smokers, ≤ 38 pack-years/>38 pack-years), and tumor histology to further explore the association between *EGFR *genotypes/haplotypes and the risk of lung cancer in each stratum. The ORs and 95% CIs in the stratification analyses were calculated using unconditional logistic regression analysis, with adjustment for gender, age or pack-years of smoking, when appropriate. The interaction between genotype and smoking was tested both with a logistic regression model that included the interaction term between genotype and smoking (pack-years of smoking or smoking exposure level), and by stratification analysis. The interaction term between genotype and smoking was not statistically significant, and this was removed from the logistic regression model. When multiple comparisons were made, the Bonferroni inequality method was used to calculate the corrected *P*-values (*Pc*-values) for multiple testing. All the analyses were performed using Statistical Analysis Software for Windows, version 8.12 (SAS institute, Gary, NC, USA).

## Results

The genotype frequencies of the *EGFR *127378C>T, 142285G>A, 162093G>A, 181946C>T and 187114T>C polymorphisms among the cases and controls and their association with lung cancer risk are shown in Table 3. The genotype distributions of the 127378C>T, 142285G>A, 162093G>A, 181946C>T and 187114T>C polymorphisms among the controls were in Hardy-Weinberg equilibrium (χ^2 ^= 0.004, *P *= 0.95; χ^2 ^= 0.98, *P *= 0.32; χ^2 ^= 1.19, *P *= 0.28; χ^2 ^= 1.26, *P *= 0.26; and χ^2 ^= 0.15, *P *= 0.70, respectively). The distribution of the 181946C>T genotypes was significantly different between the cases and controls (CC, CT and TT genotypes; 41.6%, 46.1% and 12.4% vs 36.8%, 45.9% and 17.4%; *P *= 0.04), with the frequency of the variant T allele being significantly lower in the cases than in the controls (35.5% vs 40.4%, *P *= 0.01). Compared with the 181946 CT + CC genotype, the 181946 TT genotype was associated with a significantly decreased risk of lung cancer (adjusted OR = 0.63, 95% CI = 0.45–0.88, *P *= 0.007). There was no significant difference in the genotype distributions of the other four polymorphisms studied between the cases and controls.

**Table 3:** *EGFR *genotypes of cases and controls, and their association with the risk of lung cancer

Genotypes	Cases (n = 582), no.		Controls(n = 582), no.		*P *^‡^	Crude OR (95% CI)	Adjusted OR^¶ ^(95% CI)
					
	M/F^†^	Overall(%)	M/F^†^	Overall(%)			
127378C>T							
CC	194/59	253 (43.5)	196/61	257 (44.2)	0.61	1.00	1.00
CT	208/45	253 (43.5)	216/44	260 (44.7)		0.99(0.77–1.26)	1.00(0.78–1.28)
TT	65/11	76 (13.1)	55/10	65 (11.2)		1.19(0.82–1.73)	1.22(0.83–1.78)
T allele		0.348		0.335	0.51		
142285G>A							
GG	166/40	206 (35.4)	161/54	215 (36.9)	0.80	1.00	1.00
GA	218/61	279 (47.9)	224/44	268 (46.1)		1.09(0.84–1.40)	1.11(0.86–1.44)
AA	83/14	97 (16.7)	82/17	99 (17.0)		1.02(0.73–1.44)	1.05(0.74–1.48)
A allele		0.406		0.400	0.77		
162093G>A							
GG	337/89	426 (73.2)	343/86	429 (73.7)	0.54	1.00	1.00
GA	117/26	143 (24.6)	117/28	145 (24.9)		0.99(0.76–1.30)	0.96(0.73–1.26)
AA	13/0	13 (2.2)	7/1	8 (1.4)		1.64(0.67–3.99)	1.60(0.65–3.97)
A allele		0.145		0.138	0.63		
181946C>T							
CC	195/47	242 (41.6)	181/33	214 (36.8)	0.04	1.00	1.00
CT	221/47	268 (46.1)	212/55	267 (45.9)		0.89(0.69–1.14)	0.89(0.69–1.15)
TT	51/21	72 (12.4)	74/27	101 (17.4)		0.63(0.44–0.90)*	0.59(0.41–0.85)**
CC+CT	416/94	510 (87.6)	393/88	481 (82.6)	0.02	1.00	1.00
TT	51/21	72 (12.4)	74/27	101 (17.4)		0.67(0.49–0.93)*	0.63(0.45–0.88)***,****
T allele		0.354		0.403	0.01		
187114T>C							
TT	162/42	204 (35.1)	163/42	205 (35.2)	0.78	1.00	1.00
TC	215/54	269 (46.2)	219/58	277 (47.6)		0.98(0.76–1.26)	1.01(0.78–1.31)
CC	90/19	109 (18.7)	90/15	100 (17.2)		1.10(0.79–1.53)	1.16(0.83–1.63)
C allele		0.418		0.410	0.71		

^† ^Male/Female.

^‡ ^Two-sided χ^2 ^test for either genotype distributions or allele frequencies between the cases and controls.

^¶ ^ORs (95% CIs) were calculated by conditional logistic analysis, adjusted for pack-years of smoking.

* *P *= 0.01.

** *P *= 0.005.

*** *P *= 0.007.

**** *P *= 0.32 for the interaction term between genotype and smoking status in the multivariate analysis.

The association between the *EGFR *181946C>T genotypes and the risk of lung cancer was further examined after stratification according to gender, age, smoking status, and histologic types of lung cancer. The effect of the TT genotype on the risk of lung cancer was similar in males and females, as well as in younger- and older-individuals (data not shown). When the analyses were stratified by smoking status, the protective effect of the TT genotype was statistically significant in ever-smokers (adjusted OR = 0.59, 95% CI = 0.41–0.86, *P *= 0.007; Table 4) but not in never-smokers (adjusted OR = 0.89, 95% CI = 0.45–1.75, *P *= 0.73; *P *= 0.08, test for homogeneity). Lung cancers are composed of heterogeneous histological types, and the pathways of carcinogenesis differ according to the histological types of lung cancer. Therefore, the effect of the *EGFR *181946C>T genotype on the risk of lung cancer was estimated according to the histological type of lung cancer. The protective effect of the TT genotype was pronounced in patients with small cell lung carcinoma and squamous cell carcinoma (adjusted OR = 0.32, 95% CI = 0.14–0.73, *P *= 0.007; and adjusted OR = 0.65, 95% CI = 0.41–1.01, *P *= 0.06, respectively).

**Table 4:** Stratification analysis of the *EGFR *181946C>T genotype frequencies in cases and controls

Variable	Genotype, no (%)						
		
	Cases		Controls		Adjusted OR (95% CI)		
			
	CC + CT	TT	CC+TT	TT	CC+TT	TT	*P*
Smoking status							
Never	91 (82.7)	19 (17.2)	114 (82.6)	24 (17.4)	1.0	0.89 (0.45–1.75)^†^	0.73
Ever^‡^	419 (88.8)	53 (11.2)	367 (82.7)	77 (17.3)	1.0	0.59 (0.41–0.86)^†^, *	0.007
≤ 38 pys	168 (88.4)	22 (11.6)	226 (84.0)	43 (16.0)	1.0	0.64 (0.37–1.12)^†^	0.12
> 38 pys	251 (89.0)	31 (11.0)	141 (80.6)	34 (19.4)	1.0	0.52 (0.30–0.88)^†^	0.01
Histologic types^§^							
Squamous cell ca.	238 (88.1)	32 (11.9)	481 (82.6)	101 (17.4)	1.0	0.65 (0.41–1.01)^¶^	
Adenoca.	173 (84.4)	32 (15.6)	481 (82.6)	101 (17.4)	1.0	0.82 (0.52–1.28)^¶^	
Small cell ca.	90 (92.8)	7 (7.2)	481 (82.6)	101 (17.4)	1.0	0.32 (0.14–0.73)^¶^	

^† ^Adjusted for age and pack-years of smoking.

^‡ ^Current and former smoker.

^§ ^Ten large cell carcinoma cases were excluded from this analysis.

^¶ ^Adjusted for gender and pack-years of smoking.

* *P *= 0.08, test for homogeneity test between genotype-related ORs of never- and ever-smokers.

**Figure 1:**
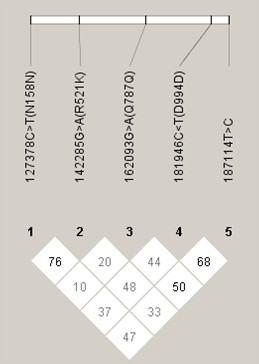
Linkage disequilibrium values |D'| (× 100) between *EGFR *polymorphisms among 582 healthy Koreans.

We estimated the *EGFR *haplotypes of the 127378C>T, 142285G>A, 162093G>A, 181946C>T, and 187114T>C polymorphisms in the cases and controls, separately, and we compared their frequency distributions between the cases and controls. The five polymorphisms were not in strong LD (Fig. 1), and thus established 29 out of the 32 (2^5^) potential haplotypes. The 25 haplotypes that had a frequency of less than 5% were pooled into a single group and included in the haplotype analysis. Table 5 shows the inferred haplotype distribution for the controls and cases, and the lung cancer risk related to each haplotype. Because the 181946TT genotype had a significant protective effect against lung cancer in the logistic regression analysis for each polymorphism, the adjusted ORs and 95% CIs were calculated using the CGGTT haplotype with the 181946T allele as the reference group. Compared to the CGGTT haplotype, the CGGCT haplotype, which is one of three haplotypes carrying the 181946C allele, was associated with a significantly increased risk of lung cancer (adjusted OR = 1.50, 95% CI = 1.09–2.07, *P *= 0.012, *Pc *= 0.048).

**Table 5:** *EGFR *haplotype frequencies in the cases and controls, and their association with lung cancer risk

Haplotype^†^	Controls (n = 1164)	Cases (n = 1164)		
		
	no. (%)	no. (%)	Adjusted^§ ^OR (95% CI)	*P*
CGGCT	91 (7.8)	118 (10.1)	1.50 (1.09–2.07)	0.012*
CGGCC	124 (10.7)	110 (9.5)	1.08 (0.80–1.47)	0.60
CGGTT	350 (30.1)	305 (26.2)	1.00	
TAGCC	227 (19.5)	234 (20.1)	1.24 (0.97–1.58)	0.09
Others^‡^	372 (32.0)	397 (34.1)	1.24 (0.98–1.54)	0.07
Global *P*		0.08		

^† ^The order of polymorphisms is as follows: 117378C>T, 142285G>A, 162093G>A, 181946C>T and 187114T>C.

^‡ ^Twenty-five haplotypes that had a frequency of less than 5%.

^§ ^Adjusted for age, gender and pack-years of smoking.

* Bonferroni corrected *P*-value = 0.048.

## Discussion

DNA sequence variations in the *EGFR *gene may lead to alteration in the production and/or activity of the EGFR, thereby causing interindividual differences in lung cancer susceptibility. To test this hypothesis, we evaluated the potential association of five *EGFR *polymorphisms (127378C>T, 142285G>A, 162093G>A, 181946C>T and 187114T>C) and the risk of lung cancer. In addition, the *EGFR *haplotypes composed of five polymorphisms were estimated, and their frequency distributions in the lung cancer cases and controls were compared. Of the five polymorphisms studied, the 181946C>T polymorphism was associated with a significantly decreased risk of lung cancer. This finding suggests that this polymorphism might be a useful marker for determining genetic susceptibility to lung cancer.

When studying polymorphisms using a limited number of subjects, low minor allele frequencies of the polymorphism may lead to null result although the polymorphism is meaningful for the risk of target disease [22-24]. In order to identify common variants (frequencies above 10%), we first determined the frequencies of the 39 reported polymorphisms in a preliminary study that included 27 healthy Koreans and 27 lung cancer cases. In the present study, we validated the presence of 11 polymorphisms in a Korean population: -540G>A, -216G>T, 2028G>A, 127378C>T, 142232C>T, 142285G>A, 151904T>A, 162093G>A, 179447T>C, 181946C>T and 187114T>C. However, the other 28 candidate polymorphisms listed in Table 1 were not detected in the preliminary study. Considering the sample size of the study, the 127378C>T, 142232C>T, 162093G>A, 181946C>T and 187114T>C that have variant allele frequencies > 10% were subjected to a case-control study to examine their association with lung cancer risk.

A few studies have investigated the association between *EGFR *polymorphisms and the risk of human cancer [25-27]. A (CA)_n _dinucleotide repeat polymorphism in intron 1 of the *EGFR *gene has been shown to affect the basal transcription activity of the *EGFR *gene; subjects with short CA repeats have been shown to have increased *EGFR *expression [28,29]. Moreover, Kang et al. [25] reported that Puerto Rican subjects having a lower number of CA repeats showed an increased risk of oral cancer. In contrast, we found that this dinucleotide polymorphism does not significantly contribute to the genetic susceptibility to lung cancer in Koreans [26], and we therefore excluded this polymorphism from the current study. In addition to the (CA)_n _repeat polymorphism, the -216G>T polymorphism, located in a Sp1 recognition site of the *EGFR *promoter, has been shown to increase the promoter activity by 30% [15]. This polymorphism has also been associated with an increased risk of glioblastoma in a European Caucasian population [27]. Despite its functional significance, the -216G>T polymorphism was not included in an association analysis in the present study because the frequency of the -216G>T polymorphism was rare (1.9%) among the 27 healthy Koreans in the preliminary study. Therefore, additional studies with larger sample sizes are needed to determine the effect of the -216G>T polymorphism on the risk of lung cancer in a Korean population.

A nonsynonymous 142285G>A (R521K) polymorphism, located in the extracellular ligand-binding domain of the *EGFR *gene has been shown to decrease ligand binding affinity, thus attenuating growth stimulation, tyrosine kinase activation and the induction of protooncogenes such as *FOS*, *JUN*, and *MYC *[13]. In the present study, however, this nonsynonymous polymorphism was not associated with the risk of lung cancer. The frequency of the variant 521K allele reported in the NIH database [17] shows remarkable variation between different ethnic groups (0.109 of African Americans, 0.250 of Caucasians, and 0.500 of Asians). Therefore, further studies are needed to clarify the association between the R521K polymorphism and lung cancer in different ethnic populations.

In the present study, individuals carrying the 181946 TT genotype were at a significantly decreased risk of lung cancer in comparison to those individuals with the 181946 CT or CC genotype. The mechanism underlying the association between the 181946C>T polymorphism and lung cancer risk remains to be elucidated. Because the 181946C>T polymorphism does not result in an amino acid change, nor does it reside within the functional domain, the observed effect of the 181946C>T polymorphism on lung cancer may be due to LD with other functional *EGFR *variant(s) that were not tested in this study. Therefore, additional studies are needed to detect the other functional variants in the *EGFR *gene and their associations with lung cancer.

Another interesting finding of this study was an interaction between the *EGFR *polymorphism and tobacco smoking. The *EGFR *181946C>T polymorphism was significantly associated with the risk of lung cancer in the smokers but not in the never-smokers, which reflects a gene-environment interaction. However, because the interaction term between the genotype and smoking was not statistically significant in the multivariate logistic regression analysis (*P *= 0.32 for the interaction term), the failure to observe a significant effect in never-smokers might be due to the relatively small number of subjects in this group. Therefore, additional studies must be conducted with a greater number of subjects in order to confirm these findings.

Recent studies have demonstrated that the haplotype has greater power to detect disease associations than an individual polymorphism on account of LD with the disease-causative variants. In addition, haplotype analysis offers the advantages of not assuming that any of the genotyped polymorphisms are functional, and it allows for the possibility of an ungenotyped functional variant to be in LD with the genotyped polymorphisms [30-32]. Therefore, our investigation was extended to analyze the *EGFR *haplotypes composed of the 127378C>T, 142285G>A, 162093G>A, 181946C>T and 187114T>C polymorphisms. In the haplotype analysis, only one (the CGGCT haplotype) of the three haplotypes carrying the 181946C allele was associated with a significantly increased risk of lung cancer in comparison to the CGGTT haplotype with the 181946T allele. These results also suggest that haplotype analysis may be a more suitable tool for assessing the disease-association than the individual polymorphism. However, the result of this haplotype analysis should be interpreted with caution due to a limitation of the computational methods used for haplotype estimation. The computational methods can be used to effectively and accurately predict haplotypes in genetic regions with pronounced LD but not in regions where marked LD is not maintained [33,34]. Therefore, since the five *EGFR *polymorphisms studied were not in strong LD, it is possible that there may have been an estimation error in the *EGFR *haplotype estimation. In addition, because this study was designed to evaluate the effects of *EGFR *polymorphisms on the risk of overall lung cancer, the stratification analyses according to age, gender, smoking status and tumor histology might have a type I error (due to multiple comparisons) and/or a type II error (due to the small number of subjects in the subgroups). Therefore, additional studies with larger sample sizes are required to confirm our findings.

## Conclusion

In this study, we tested the hypothesis that polymorphisms in the *EGFR *gene can affect the risk of lung cancer in the general population. We found that the 181946C>T polymorphism was associated with the risk of lung cancer. This result suggests that the *EGFR *181946C>T polymorphism could be used as a marker for the genetic susceptibility to lung cancer; however, additional studies with larger sample sizes are needed to confirm our findings. Future studies on the other *EGFR *sequence variants and their biological function are also needed in order to understand the role of the 181946C>T polymorphism in determining the risk of lung cancer. Moreover, because genetic polymorphisms often vary between different ethnic groups, further studies are needed to clarify the association of the *EGFR *polymorphisms with the risk of lung cancer in diverse ethnic populations.

## Abbreviations used

EGFR, epidermal growth factor receptor; SNP, single nucleotide polymorphisms; LD, linkage disequilibrium; OR, odds ratio; CI, confidence interval; *Pc*-value, Bonferroni corrected *P*-value.

## Competing interests

The author(s) declare that they have no competing interests.

## Authors' contributions

JEC, SHP and KMK participated in the design of study, and carried out sequencing and genotyping, and participated in statistical analysis and interpretation of data. WKL and SK participated in the design of study, and analysis and interpretation of data. SIC, CHK, THJ participated in the design of study and collected clinical data. YMK, YCK and SBH participated in the design of study and helped to draft the manuscript. JYP conceived the study and participated in the design of study, analysis and interpretation of data, drafting the article and final approval of this version. All authors read and approved the final manuscript.

## Pre-publication history

The pre-publication history for this paper can be accessed here:


